# Understanding the mechanisms of lateral parietal memory modulation in Alzheimer's Disease

**DOI:** 10.1002/alz.093640

**Published:** 2025-01-09

**Authors:** Matthew A Slayton, Margaret L McAllister, Jane Rothrock, Sarrah Belhadj, Kirsten D Gillette, Mariam Hovhannisyan, Roberto Cabeza, Simon W Davis

**Affiliations:** ^1^ Duke University, Durham, NC USA; ^2^ UNC, Chapel Hill, NC USA; ^3^ University of Arizona, Tucson, AZ USA

## Abstract

**Background:**

Alzheimer’s Disease (AD) is characterized by progressive impairment of cognition and memory, including the loss of episodic memory. The use of non‐invasive brain stimulation therapies to modulate memory encoding processes is a promising avenue for potential treatment. Previous studies have shown that the use of Transcranial Magnetic Stimulation (TMS) applied to lateral parietal cortex can improve memory in older adults who have received a diagnosis of Mild Cognitive Impairment. This effect may be driven by the modulation of connectivity between parietal cortex and the hippocampus, structures which support the encoding of semantic information into memory. And yet, while modulation of cortical‐hippocampal connectivity helps to establish a systems‐level explanation for the parietal memory benefit for TMS, it does not explain the benefit on the level of memory representation—i.e., the most basic evidence we have of what visual and semantic information underlies memory formation. Previous work from our lab has found that TMS can change memory representations in the hippocampus.

**Method:**

Here we present new data from a study applying this approach to MCI, using rTMS to an individualized site in parietal cortex to boost memory function, while monitoring daily changes in behavior and brain physiology over the course of three days. Immediately after rTMS, patients perform a semantic encoding task where they view everyday objects while fMRI is collected, after which they complete recognition memory tasks. To ask whether memory performance improvements are driven by the modulation of visual or semantic information in the brain, we use Representational Similarity Analysis (RSA), a computational method that can reveal the changes in visual and semantic representations over the course of TMS.

**Result:**

Consistent with the role of lateral parietal cortex as a hub for the processing of abstract knowledge, we show that semantic representations show greater TMS‐related changes and are more directly associated with improvements in memory success, whereas visual representations are not.

**Conclusion:**

Increasing the representation of abstract knowledge is a potential underlying mechanisms by which neuromodulation boosts episodic memory in MCI, which may lead to reliable clinical applications of noninvasive brain stimulation in AD.

